# 初诊危重急性髓系白血病的临床特征及预后分析

**DOI:** 10.3760/cma.j.cn121090-20241211-00561

**Published:** 2025-01

**Authors:** 佩淇 梁, 梦 高, 妍 谢, 冰清 李, 倩 李, 子溢 刘, 栋 王, 惠英 仇, 苏宁 陈, 德沛 吴, 建红 付

**Affiliations:** 国家血液系统疾病临床医学研究中心，苏州大学附属第一医院血液科重症监护病房，江苏省血液研究所，苏州 215006 National Clinical Research Center for Hematologic Diseases, the First Affiliated Hospital of Soochow University, Department of Hematology, Intensive Care Unit, Jiangsu Institute of Hematology, Suzhou 215006, China

**Keywords:** 白血病，髓系，急性, 重症监护病房, 危重, 早期死亡, Leukemia, myeloid, acute, Intensive care unit, Critical illness, Early death

## Abstract

**目的:**

回顾性分析初诊危重急性髓系白血病（AML）收住血液专科重症监护病房（HCU）患者的临床特征，分析AML危重症出现的特点和早期死亡相关因素。

**方法:**

收集2020年10月至2024年10月苏州大学附属第一医院HCU收治的91例初诊AML患者的临床资料，分析患者收住HCU的原因及HCU的主要治疗措施，分析危重患者早期死亡的危险因素。

**结果:**

初诊AML患者从诊断至入HCU的中位时间为3（3, 9）d，中位住HCU天数为10（3, 23）d，71例患者在诱导化疗前收住HCU，20例在诱导化疗开始后转入HCU。收入HCU的最主要原因为肺部感染（78.0％），其次分别是呼吸衰竭（44.0％）、肝功能不全（28.6％）、肾功能不全（27.5％）、弥散性血管内凝血（DIC）（25.3％）和脓毒症（23.1％），入HCU时中位急性生理与慢性健康Ⅱ评分（APACHE Ⅱ评分）14（11, 18）分，中位脓毒症序贯器官衰竭评分（SOFA评分）7（4, 10）分，HCU的主要救治措施包括血管活性药物、无创机械通气、连续性肾脏替代治疗、有创机械通气及治疗性白细胞清除。接受诱导化疗的患者复合完全缓解率及总体缓解率分别为65.4％和88.5％。35例（38.5％）患者在入HCU后28 d内死亡，DIC（*OR*＝9.350，95％ *CI* 1.999～43.745，*P*＝0.005）、脓毒症（*OR*＝6.817，95％ *CI* 1.571～29.582，*P*＝0.010）及心功能不全（*OR*＝12.281，95％ *CI* 2.385～63.254，*P*＝0.003）是患者28 d死亡的独立危险因素。

**结论:**

初诊危重AML患者入住HCU的主要原因为肺部感染，近四成患者早期死亡，DIC、脓毒症及心功能不全是影响早期死亡的因素。

## Abstract (en)

**Objective:**

This study retrospectively analyzed the clinical characteristics of patients newly diagnosed with acute myeloid leukemia（AML）who were admitted to the hematology intensive care unit（HCU）with critical illness. It also examined factors associated with critical illness and early mortality in these patients.

**Methods:**

Clinical data were collected from 91 newly diagnosed AML patients admitted to the HCU of the Department of Hematology, First Affiliated Hospital of Soochow University, from October 2020 to 2024. Reasons for HCU admission, major therapeutic interventions, and risk factors for critical illness and early mortality were analyzed.

**Results:**

The median time from diagnosis to HCU admission was 3 days（*IQR*: 3–9 days）, and the median HCU stay was 10 days（*IQR*: 3–23 days）. Of the 91 patients, 71 were admitted to the HCU before induction chemotherapy, while 20 were transferred to the HCU after its initiation. The leading causes of HCU admission were pulmonary infection（78.0％）, respiratory failure（44.0％）, hepatic insufficiency（28.6％）, renal insufficiency（27.5％）, disseminated intravascular coagulation（DIC; 25.3％）, and sepsis（23.1％）. Median Acute Physiology and Chronic Health Evaluation Ⅱ（APACHE Ⅱ）and SOFA scores at HCU admission were 14（*IQR*: 11–18）and the median Sepsis Related Organ Failure Assessment（SOFA）score was 7（*IQR*: 4, 10）. Major HCU interventions included vasoactive drugs, noninvasive and invasive mechanical ventilation, continuous renal replacement therapy, therapeutic leukocyte clearance, and cardiopulmonary resuscitation. Among patients receiving induction chemotherapy, the composite complete remission rate was 65.4％, and the overall remission rate was 88.5％. Thirty-five（38.5％）patients died within 28 days of HCU admission. Independent risk factors for 28-day mortality were DIC（*OR*＝9.350, 95％ *CI* 1.999–43.745，*P*＝0.005）, sepsis（*OR*＝6.817, 95％ *CI* 1.571–29.582, *P*＝0.010）, and cardiac insufficiency（*OR*＝12.281, 95％ *CI* 2.385–63.254, *P*＝0.003）.

**Conclusion:**

The main reason for HCU admission in newly diagnosed critically ill AML patients was pulmonary infection. Nearly 40％ of patients experisenced early death, and DIC, sepsis, and heart failure were factors influencing early mortatlity.

未经治疗的初诊急性髓系白血病（AML）患者容易出现感染、出血、白细胞瘀滞、弥散性血管内凝血（DIC）、脏器功能不全等并发症。约4.1％ AML患者在诊断后1个月内死亡，部分患者甚至在诊断后3 d内死亡，10％～15％的患者会出现严重的不良事件。因此尽早识别危重AML患者，及时给予适当的重症监护病房（ICU）支持治疗对改善预后具有重要的意义。部分初诊AML患者诱导化疗期间患者会出现肿瘤溶解综合征（Tumor lysis syndrome, TLS）、呼吸衰竭、脓毒症等危及生命的急重症并发症。危重AML患者对ICU的需求逐年升高[1]，加拿大一项大样本队列研究表明约22％的AML患者在确诊后第1年内需要入住ICU救治[2]。本研究回顾性分析了收住血液重症监护病房（HCU）的初诊AML患者的临床特征及早期救治情况，现报道如下。

## 病例与方法

1. 病例资料：本研究回顾性分析苏州大学附属第一医院HCU 2020年10月至2024年10月诊治的91例初诊AML［非急性早幼粒细胞白血病（APL）］患者的临床资料，所有患者诊断均符合《成人急性髓系白血病（非急性早幼粒细胞白血病）中国诊疗指南（2023年版）》标准[3]。

2. 临床资料收集：①一般资料：包括性别，年龄，入ICU时的血常规、总胆红素、转氨酶、肌酐、血清乳酸脱氢酶（LDH）、肌钙蛋白等实验室指标。②骨髓检查结果及化疗资料：患者诊断时骨髓原始细胞比例、白血病免疫分型、染色体、融合基因、突变基因等实验室指标，化疗药物使用及疾病缓解情况等临床资料，患者细胞遗传学/分子遗传学指标危险度分级遵循ELN2022 AML指南标准[4]。③HCU评估：全部患者入HCU即刻完成生命体征检测、脓毒症序贯器官衰竭评分（Sepsis Related Organ Failure Assessment, SOFA）及急性生理与慢性健康Ⅱ（Acute Physiology and Chronic Health Evaluation Ⅱ, APACHE Ⅱ）评分，因本研究病例均为AML患者，常因骨髓抑制或恶性浸润问题存在血小板减少，故我们在进行SOFA评分时剔除了血小板部分。④本研究为短期预后分析，评估患者的骨髓缓解情况，并以随访患者28 d的生存结局作为本研究主要终点，评价其预后。

3. 化疗方案：①传统化疗方案：包括IA（去甲氧柔红霉素+阿糖胞苷）、HA（高三尖杉酯碱+阿糖胞苷）、地西他滨+HAAG（高三尖杉酯碱+阿糖胞苷+阿克拉霉素+G-CSF）、IAG（去甲氧柔红霉素+阿糖胞苷+G-CSF）方案；②VEN+HMA（维奈克拉联合去甲基化药物）方案：维奈克拉联合地西他滨或阿扎胞苷，部分患者再加用其他靶向药物如吉瑞替尼。

4. 相关定义：高白细胞血症：WBC≥100.0×10^9^/L；完全缓解（CR）：骨髓原始细胞比例<5％，外周血未见原始细胞，无髓外病变，ANC≥1.0×10^9^/L且PLT≥100×10^9^/L；CR伴血细胞不完全恢复（CRi）：骨髓原始细胞比例<5％，外周血未见原始细胞，无髓外病变，ANC<1.0×10^9^/L或PLT<100×10^9^/L；复合CR（cCR）：CR+CRi；部分缓解（PR）：血细胞恢复达CR，骨髓原始细胞比例较初始值降幅≥50％；未缓解（NR）：未达到cCR或PR；总缓解率（ORR）：cCR率+PR率。

5. 统计学处理：应用SPSS 26.0软件进行统计学分析。分类变量以例数（构成比）进行描述，组间比较采用卡方检验或Fisher精确概率法；连续变量以均数±标准差（服从正态分布）或中位数（*Q*_1_，*Q*_3_）（不服从正态分布）进行描述。对可能影响28 d死亡的因素进行单因素Logistics回归分析，单因素*P*<0.05的变量纳入多因素Logistic回归分析。双侧*P*<0.05为差异有统计学意义。

## 结果

1. 患者临床特征：91例收HCU的危重初诊AML（非APL）患者临床特征详见表1。男49例，女42例，中位年龄53（16～83）岁，60岁及以上患者36例（39.6％）。71例（78.0％）患者在诱导化疗启动前（早期）收住ICU，20例（22.0％）在诱导化疗启动后（晚期）转入。原发AML患者有87例，骨髓增生异常综合征转化的AML患者4例。根据形态学FAB分型，M_0_ 1例，M_1_ 8例，M_2_ 27例，M_4_ 10例，M_5_ 35例，M_7_ 1例，9例不能分类。骨髓细胞遗传学与分子遗传学危险分层：除外5例在检验结果报告前死亡故未能分类（5.5％），低危组14例（15.4％），中危组26例（28.6％），高危组46例（50.5％），其中突变发生频率前4位的分别是FLT3（28.6％）、NPM1（17.6％）、TP53（14.3％）及DNMT3A（14.3％），融合基因发生频率最高的是CBFβ∷MYH11（12.1％）。入HCU时中位WBC为28.18（3.51, 85.31）×10^9^/L，中位LDH为829.1（386.5, 1 797.5）U/L，中位骨髓原始细胞比例为64.8％（41％, 84％）。从诊断至入HCU的中位时间为3（3, 9）d，中位HCU住院天数为10（3, 23）d。

**表1:** 91例危重急性髓系白血病患者的临床特征

临床特征	数值
年龄［岁，*M*（*Q*_1_, *Q*_3_）］	53.0（41.0, 65.5）
性别（例，男/女）	49/42
疾病类型［例（％）］	
原发性	87（95.6）
MDS转化	4（4.4）
FAB分型［例（％）］	
M_0_	1（1.1）
M_1_	8（8.8）
M_2_	27（29.7）
M_4_	10（11.0）
M_5_	35（38.5）
M_7_	1（1.1）
不能分类	9（9.9）
ELN危险度分层［例（％）］	
低危	14（15.4）
中危	26（28.6）
高危	46（50.5）
不能分类	5（5.5）
基因突变［例（％）］	
FLT3	26（28.6）
NPM1	16（17.6）
TP53	13（14.3）
DNMT3A	13（14.3）
IDH2	10（11.0）
CEBPA	9（9.9）
ASXL1	8（8.8）
RUNX1	6（6.6）
BCOR	4（4.4）
初诊时骨髓原始细胞比例［％，*M*（*Q*_1_, *Q*_3_）］	65（41, 84）
入HCU时WBC［×10^9^/L，*M*（*Q*_1_, *Q*_3_）］	28.18（3.51, 85.31）
入HCU时LDH［U/L，*M*（*Q*_1_, *Q*_3_）］	829.1（386.5, 1 797.5）
APACHE Ⅱ评分［*M*（*Q*_1_, *Q*_3_）］	14（11, 18）
SOFA评分［*M*（*Q*_1_, *Q*_3_）］	6（4, 10）
诊断至入HCU时间［d，*M*（*Q*_1_, *Q*_3_）］	3（3, 9）
HCU住院天数［d，*M*（*Q*_1_, *Q*_3_）］	10（3, 23）

**注** MDS：骨髓增生异常综合征；ELN：欧洲白血病协作网；HCU：血液重症监护病房；APACHE Ⅱ评分：急性生理与慢性健康Ⅱ评分；SOFA评分：脓毒症序贯器官衰竭评分

初诊AML患者收入HCU的主要原因为肺部感染（78.0％），其次分别是呼吸衰竭（44.0％）、肝功能不全（28.6％）、肾功能不全（27.5％）、DIC（25.3％）、脓毒症（23.1％）、心功能不全（23.1％）、高白细胞血症（20.9％）、弥漫性肺泡出血（Diffuse alveolar hemorrhage, DAH）（19.8％）、TLS（16.5％）、脑出血（9.9％）、急性冠脉综合征（5.5％）及消化道出血（3.3％）。诱导化疗前进入HCU原因前3位分别是肺部感染、呼吸衰竭、DIC，而诱导化疗开始后入HCU原因前3位分别是肺部感染、呼吸衰竭、脓毒症，其中诱导开始后组脓毒症的患者比例显著高于诱导前组，而诱导前组高白细胞血症的患者比例显著高于诱导开始后组（*P*<0.05）。入HCU时中位APACHE Ⅱ评分14（11, 18）分，中位SOFA评分7（4, 10）分。HCU的主要救治措施包括血管活性药物（34.1％）、无创机械通气（28.6％）、连续性肾脏替代治疗（CRRT）（24.2％）、有创机械通气（22.0％）、治疗性白细胞清除（19.8％）及心肺复苏（11.0％）。诱导前入HCU组救治措施前3位分别是血管活性药物、无创机械通气及治疗性白细胞清除，而诱导开始后入HCU组救治措施前3位分别是CRRT、血管活性药物及无创机械通气（表2）。

**表2:** 危重急性髓系白血病患者收住HCU的病因及治疗措施（例数）

病因及治疗措施	总体（91例）	早期收入HCU（71例）	晚期收入HCU（20例）	*P*值
并发症				
肺部感染	71	56	15	0.763
呼吸衰竭	40	29	11	0.312
肝功能不全	26	19	7	0.579
肾功能不全	25	19	6	0.782
DIC	23	21	2	0.088
脓毒症	21	10	11	<0.001
心功能不全	21	17	4	0.485
高白细胞血症	19	19	0	0.010
DAH	19	13	6	0.213
TLS	15	11	4	0.734
脑出血	9	8	1	0.678
急性冠脉综合征	5	5	0	0.582
消化道出血	3	3	0	1.000
ICU治疗措施				
血管活性药物	31	25	6	0.792
无创机械通气	26	20	6	1.000
CRRT	22	14	8	0.078
有创机械通气	20	16	4	1.000
白细胞清除	18	18	0	0.010

**注** HCU：血液重症监护病房；DIC：弥散性血管内凝血；DAH：弥漫性肺泡出血；TLS：肿瘤溶解综合征；CRRT：连续性肾脏替代治疗

2. 诱导化疗：71例（78.0％）患者在诱导化疗启动前收住HCU，其中有45例在HCU接受诱导化疗，3例接受传统化疗方案，42例接受VEN+HMA方案。20例（21.9％）患者在诱导化疗启动后转入HCU，11例接受传统化疗方案，9例接受VEN+HMA方案。全部65例接受诱导化疗的患者中有52例完成了诱导化疗，cCR率及ORR分别为65.4％和88.5％，传统化疗方案组（14例）和VEN+HMA方案组（38例）的cCR率分别为50.0％和71.1％，差异无统计学意义（*P*＝0.197）。有34例患者全程在HCU完成诱导化疗，其cCR率及ORR率分别为76.5％和97.1％。

3. 死亡危险因素：35例（38.5％）患者在入HCU后28 d内死亡，其中24例（26.4％）患者在7 d内死亡。早期入ICU组患者28 d死亡率为38.0％，晚期入HCU组28 d死亡率为40.0％，差异无统计学意义（*P*＝1.000）。91例患者中有65例接受了诱导化疗，52例完成了诱导化疗，13例在诱导过程中死亡。我们对完成化疗的52例患者进行分析发现cCR、PR、NR组28 d死亡率分别为5.9％（2/34）、16.7％（2/12）和50.0％（3/6），差异有统计学意义（*P*＝0.015）。死亡组患者的年龄、APACHE Ⅱ评分、SOFA评分均高于非死亡组患者，死亡组患者的呼吸衰竭、DIC、脓毒症、心功能不全、肾功能不全、TLS及DAH的比例均高于存活组，死亡组使用血管活性药物、无创机械通气、CRRT及有创机械通气的比例均高于存活组，详见表3。将年龄、呼吸衰竭、DIC、脓毒症、心力衰竭、肾功能不全、TLS、DAH进行多因素Logistic回归分析发现，患者存在DIC、脓毒症及心功能不全是28 d死亡的危险因素，详见表4。

**表3:** 危重急性髓系白血病患者收住HCU 28 d死亡单因素Logistic回归分析

变量	*OR*（95％ *CI*）	*P*值
年龄	1.03（1.01 ～ 1.06）	0.044
性别（女）	1.41（0.60 ～ 3.30）	0.426
骨髓原始细胞比例	0.61（0.10 ～ 3.63）	0.585
LDH	1.00（1.00 ～ 1.00）	0.233
ELN危险度分层		
低危	参照	
中危	1.11（0.27 ～ 4.63）	0.885
高危	1.61（0.44 ～ 5.91）	0.475
诱导化疗后入HCU	1.09（0.39 ～ 3.00）	0.873
APACHEⅡ评分	1.12（1.03 ～ 1.20）	0.005
SOFA评分	1.44（1.23 ～ 1.69）	<0.001
高白细胞	0.50（0.16 ～ 1.54）	0.227
肺部感染	0.54（0.20 ～ 1.48）	0.233
呼吸衰竭	5.45（2.17 ～ 13.68）	<0.001
脓毒症	6.25（2.12 ～ 18.39）	<0.001
DIC	4.50（1.65 ～ 12.28）	0.003
消化道出血	3.33（0.29 ～ 38.20）	0.333
肝功能不全	1.95（0.78 ～ 4.93）	0.155
心功能不全	8.59（2.76 ～ 26.70）	<0.001
肾功能不全	3.45（1.32 ～ 8.98）	0.011
TLS	4.08（1.26 ～ 13.22）	0.019
脑出血	1.32（0.33 ～ 5.28）	0.698
DAH	4.35（1.45 ～ 13.03）	0.009

**注** HCU：血液重症监护病房；ELN：欧洲白血病协作网；APACHE Ⅱ评分：急性生理与慢性健康Ⅱ评分；SOFA评分：脓毒症序贯器官衰竭评分；DIC：弥散性血管内凝血；TLS：肿瘤溶解综合征；DAH：弥漫性肺泡出血

**表4:** 危重急性髓系白血病患者收住HCU 28 d死亡多因素Logistic回归分析

变量	*OR*（95％ *CI*）	*P*值
年龄	0.986（0.946 ～ 1.027）	0.492
呼吸衰竭	3.382（0.880 ～ 13.002）	0.076
脓毒症	6.817（1.571 ～ 29.582）	0.010
DIC	9.350（1.999 ～ 43.745）	0.005
心功能不全	12.281（2.385 ～ 63.254）	0.003
肾功能不全	0.612（0.082 ～ 4.584）	0.633
TLS	6.815（0.572 ～ 81.138）	0.129
DAH	2.833（0.613 ～ 13.092）	0.183

**注** HCU：血液重症监护病房；DIC：弥散性血管内凝血；TLS：肿瘤溶解综合征；DAH：弥漫性肺泡出血

## 讨论

既往研究发现大出血、肝功能衰竭、肾功能衰竭、呼吸衰竭和心脏骤停与AML患者早期（60 d内）死亡相关，高龄、低血红蛋白水平和低格拉斯哥评分与极早期（30 d内）死亡显著相关[5]–[6]。我们的研究发现，未治疗的AML患者入HCU的主要原因前3位分别是肺部感染、呼吸衰竭和DIC，而诱导化疗开始后入HCU原因前三位分别是肺部感染、呼吸衰竭和脓毒症，这可能与诱导化疗后患者由于更深的骨髓抑制更容易发生重症感染相关。AML患者在HCU接受最多的救治措施包括血管活性药物、无创机械通气和CRRT。不适合高强度化疗的初治AML患者常选用维奈克拉联合去甲基化药物方案或减低剂量化疗等，危重初诊患者的化疗方案选择更应慎重，需兼顾疗效和安全性[7]–[8]。我们前期研究发现危重初诊AML患者使用维奈克拉联合去甲基化药物方案CR/CRi率为75％，总体安全性尚可[9]。本研究纳入的患者cCR率为65.4％，VEN+HMA方案组的缓解率高于传统化疗方案组，但差异无统计学意义。Ahmed等[10]2017年报道的AML转入ICU的患者中位总生存期为1.3个月，ICU死亡率为61％，急性呼吸衰竭是转入ICU的最常见原因，机械通气、血管活性药物和肾脏替代治疗是AML患者在ICU常用支持手段。Lengliné等[11]研究发现早期ICU住院治疗可以避免脏器功能衰竭，晚期ICU入院的患者入院时血压和血氧饱和度降低，机械通气和CRRT使用率升高，ICU住院时间延长，ICU生存率降低。我们的研究发现患者DIC、脓毒症和心功能不全是患者早期死亡的独立危险因素。因此，早期识别危重患者，在出现脏器功能衰竭前收住ICU进行严密监测及器官功能支持治疗，可以避免危及生命的并发症发生，改善AML患者的预后。

TLS是由于大量肿瘤细胞裂解破坏后释放其内容物，所导致的一组代谢异常、引起机体多器官损伤的临床综合征，最常见于治疗开始后1周左右的时间，也可见于治疗开始前，严重时迅速进展可危及生命[12]。研究报道AML患者使用维奈克拉的TLS发生率为1.1％～12％[13]–[14]。我们的研究中有15例患者发生了TLS，9例发生在应用维奈克拉之后，2例发生在强化疗后，3例在羟基脲/小剂量阿糖胞苷降白细胞过程中，1例为自发性，14例TLS患者应用了CRRT治疗。随着维奈克拉在临床上的应用渐广，TLS的发生率逐渐增加，而肾功能损伤严重影响患者的预后，因此临床医师在用药前应加强预防，严格遵循维奈克拉的剂量爬坡给药原则，必要时也可从小剂量开始应用。既往研究认为伴有NMP1或IDH2突变的AML患者使用维奈克拉时更易发生TLS[15]。我们的研究发现，发生TLS的患者中FLT3突变有3例，TP53突变2例，DNMT3A突变2例，NPM1和IDH2突变各1例，未来需要更多的病例和研究来探究其相关性。

DIC通常被认为是APL患者的特征性并发症，研究显示70％～80％的APL病例和高达10％～32％的非APL-AML病例存在DIC[16]。在接受强化化疗的AML患者中，出血是导致早期死亡的主要原因[17]。既往曾有报道初诊的AML约有21％的病例出现明显DIC，并且与高龄、合并症、体能状态不佳、白细胞增多、LDH水平、NPM1突变、CD33和CD4表达以及CD34表达缺失有关。AML患者中存在NPM1或FLT3-ITD突变被认为DIC风险增加，此外，KMT2A基因重排的AML患者出血和DIC的发生率更高[18]–[19]。我们的报道中危重AML患者DIC的发生率为25.3％，诱导前DIC发生率为29.6％，诱导开始后DIC发生率为10％，23例DIC患者中FLT3突变有8例（34.8％），所占比例最高，其次是NPM1突变（17.4％）和TP53突变（17.4％）。DIC是初诊AML患者早期死亡的独立危险因素，早期识别并干预患者的凝血障碍可能有助于改善患者的预后。

初诊AML患者常会出现危及生命的特殊急重并发症，这些初诊相关危重症所致的早期死亡仍是目前AML治疗的巨大挑战。我们中心从2020年10月建立HCU，探索早期识别具有高危特征的初诊AML收入HCU进行临床集中管理，能较好结合血液专科诊疗手段和ICU综合支持技术，及时进行AML精准诊断和危险度分层，并且在清洁病房严格防护条件下及时进行AML个体化诱导治疗，由HCU专业医护团队对危重并发症进行有针对性的预防和抢先干预，我们的经验表明HCU救治模式有利于改善初诊危重AML的生存预后。

## References

[1] Röllig C, Kramer M, Schliemann C (2020). Does time from diagnosis to treatment affect the prognosis of patients with newly diagnosed acute myeloid leukemia?[J]. Blood.

[2] Ferreyro BL, Scales DC, Wunsch H (2021). Critical illness in patients with hematologic malignancy: a population-based cohort study[J]. Intensive Care Med.

[3] 中华医学会血液学分会白血病淋巴瘤学组 (2023). 成人急性髓系白血病(非急性早幼粒细胞白血病)中国诊疗指南(2023年版)[J]. 中华血液学杂志.

[4] Döhner H, Wei AH, Appelbaum FR (2022). Diagnosis and management of AML in adults: 2022 recommendations from an international expert panel on behalf of the ELN[J]. Blood.

[5] Ho G, Jonas BA, Li Q (2017). Early mortality and complications in hospitalized adult Californians with acute myeloid leukaemia[J]. Br J Haematol.

[6] Kusuda M, Nakasone H, Nakamura Y (2023). Very early death within 30 days after diagnosis in patients with acute myeloid leukemia[J]. Int J Hematol.

[7] 孙 立, 叶 少杰, 周 楠 (2022). 维奈克拉联合阿扎胞苷治疗不适合标准化疗的新诊断急性髓系白血病疗效分析:单中心数据[J]. 中华血液学杂志.

[8] Miyamoto T, Sanford D, Tomuleasa C (2022). Real-world treatment patterns and clinical outcomes in patients with AML unfit for first-line intensive chemotherapy[J]. Leuk Lymphoma.

[9] Liang P, Xie Y, Liu Z (2024). Venetoclax and hypomethylating agents in critically ill patients with newly diagnosed acute myeloid leukaemia[J]. Br J Haematol.

[10] Ahmed T, Koch AL, Isom S (2017). Outcomes and changes in code status of patients with acute myeloid leukemia undergoing induction chemotherapy who were transferred to the intensive care unit[J]. Leuk Res.

[11] Lengliné E, Raffoux E, Lemiale V (2012). Intensive care unit management of patients with newly diagnosed acute myeloid leukemia with no organ failure[J]. Leuk Lymphoma.

[12] Durani U, Hogan WJ (2020). Emergencies in haematology: tumour lysis syndrome[J]. Br J Haematol.

[13] DiNardo CD, Jonas BA, Pullarkat V (2020). Azacitidine and Venetoclax in Previously Untreated Acute Myeloid Leukemia[J]. N Engl J Med.

[14] Shahswar R, Beutel G, Gabdoulline R (2021). Risk of tumor lysis syndrome in patients with acute myeloid leukemia treated with venetoclax-containing regimens without dose ramp-up[J]. Ann Hematol.

[15] DiNardo CD, Wei AH (2020). How I treat acute myeloid leukemia in the era of new drugs[J]. Blood.

[16] Wang TF, Makar RS, Antic D (2020). Management of hemostatic complications in acute leukemia: Guidance from the SSC of the ISTH[J]. J Thromb Haemost.

[17] Versluis J, Pandey M, Flamand Y (2022). Prediction of life-threatening and disabling bleeding in patients with AML receiving intensive induction chemotherapy[J]. Blood Adv.

[18] Guo Z, Chen X, Tan Y (2020). Coagulopathy in cytogenetically and molecularly distinct acute leukemias at diagnosis: Comprehensive study[J]. Blood Cells Mol Dis.

[19] Nguyen D, Kantarjian HM, Short NJ (2023). Early mortality in acute myeloid leukemia with KMT2A rearrangement is associated with high risk of bleeding and disseminated intravascular coagulation[J]. Cancer.

